# Primary central nervous system angiosarcoma: two case reports

**DOI:** 10.1186/1752-1947-6-251

**Published:** 2012-08-21

**Authors:** James R Hackney, Cheryl Ann Palmer, Kristen O Riley, Joel K Cure, Hassan M Fathallah-Shaykh, Louis B Nabors

**Affiliations:** 1 Division of Neuropathology, University of Alabama at Birmingham, PD6A 175, Birmingham, AL, 35294, USA; 2 Department of Neurology, University of Alabama at Birmingham, FOT 1020, Birmingham, AL, 35294, USA; 3 Division of Neurosurgery, University of Alabama at Birmingham, FOT 1046, Birmingham, AL, 35294, USA; 4 Department of Radiology, University of Alabama at Birmingham, JT N418, Birmingham, AL, 35294, USA

**Keywords:** Angiosarcoma, Primary Central Nervous System (CNS) Tumors, Sarcoma, Bevacizumab, Temozolomide

## Abstract

**Introduction:**

Primary angiosarcoma of the brain is extremely rare; only 15 cases have been reported in adults over the last 25 years.

**Case presentations:**

We describe two cases of primary angiosarcoma of the brain that are well characterized by imaging, histopathology, and immunohistochemistry. Case 1: our first patient was a 35-year-old woman who developed exophthalmos. Subtotal resection of a left extra-axial retro-orbital mass was performed. Case 2: our second patient was a 47-year-old man who presented to our facility with acute visual loss, word-finding difficulty and subtle memory loss. A heterogeneously-enhancing left sphenoid wing mass was removed. We also review the literature aiming at developing a rational approach to diagnosis and treatment, given the rarity of this entity.

**Conclusions:**

Gross total resection is the standard of care for primary angiosarcoma of the brain. Adjuvant radiation and chemotherapy are playing increasingly recognized roles in the therapy of these rare tumors.

## Introduction

Primary angiosarcoma of the central nervous system (CNS) is an extremely rare malignancy, with only approximately 15 cases reported in adults in the literature over the last 25 years. Mena *et al.*[1] contributed six cases in adults, the total number of cases from the files of the Armed Forces Institutes of Pathology (AFIP) between 1970 to 1987. Merimsky *et al.*[2] reported a small series of CNS sarcomas that included three angiosarcomas, while Paulus *et al.*[3] also reported a slightly larger series of primary intra-cranial sarcomas that included two angiosarcomas. The remaining cases derive from single case reports [4-9] (Table 1).

**Table 1:** Angiosarcoma of the brain in adults: case reports and series

**Series**	**Sex**	**Age**	**Location**	**Therapy**	**Survival**
Charman *et al*. [5]	M	65	Occipital	Surgery	12 months
Paulus *et al*. [3]	M; F	24; 26	Temporal; frontal	Unknown	Unknown
Mena *et al*. [1]	M (5), F (3)	2 weeks to 72 years (mean 38 years)	Hemisphere (6), meninges (1), unknown (1)	Surgery	Four months (4), 30 months (1), alive 39 months (1), alive 102 months (1), unknown (1)
Cookston *et al*. [6]	F	32	Occipital	Surgery + RT	Alive three-and-a-half years
Fuse *et al*. [7]	M	39	Parietal	Surgery + RT	29 months
Antoniadis *et al*. [4]	M	41	Parietal	Surgery + RT + chemotherapy	Alive 41 months
Lach and Benoit [9]	M	37	Frontoparietal	Surgery	18 months
Merimsky *et al*. [2]	M; M	27; 18	Parietal; occipitoparietal	Radiation only; surgery	Two-and-a-half months; five months

The study of Mena *et al*. includes two pediatric cases whose data are entangled with the adult cases. These two cases are not included in our discussion.

*RT* radiation therapy.

Charman *et al*. [5] described the first case of primary CNS angiosarcoma in an adult; a 65-year-old man, treated by surgical resection followed by radiation therapy, who survived for 13 months. Since this initial report, the hallmarks of CNS angiosarcoma are recognized as highly vascular malignant tumors with aggressive local recurrence and only modest response to chemotherapy or radiation treatment [10].

We report two cases of primary CNS angiosarcoma well characterized by imaging, histopathology, and immunohistochemistry. We discuss these cases in the context of similar cases reported within the last 35 years in an attempt to develop a rational approach to diagnosis and treatment, given the rarity of this entity.

## Case presentations

### Patient 1

**Figure 1:**
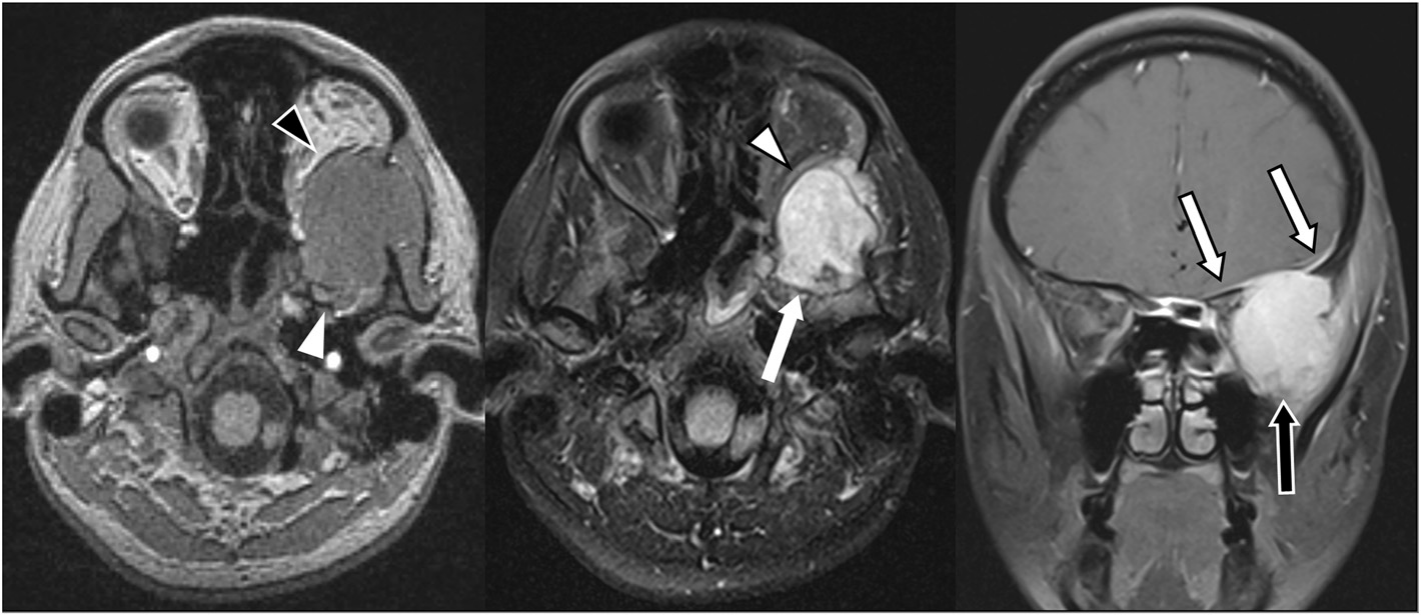
**Axial unenhanced T1-weighted magnetic resonance image demonstrating a large mass in the left middle fossa (white arrowhead) extending through the left greater sphenoid wing into the left orbit (outlined black arrowhead).** The fat-suppressed axial T1-weighted gadolinium-diethylenetriaminepentaacetic acid (Gd-DTPA)-enhanced image demonstrates the same findings (white arrow, outlined white arrowhead, respectively). The mass is enhanced homogeneously. The coronal fat-suppressed axial T1-weighted Gd-DTPA-enhanced image demonstrates the left middle fossa component (outlined black arrow) and extension up and over the margin of the left sphenoid ridge producing dural thickening and enhancement here (outlined white arrows).

A 35-year-old Caucasian woman presented to an outside facility with left eye irritation. Over the next six months, her symptoms progressed to exophthalmos with blurred vision. Imaging studies demonstrated an extra-axial homogeneous mass of the left retro-orbital infra-temporal area compressing the temporal lobe and displacing the left optic nerve. There was homogeneous enhancement of the mass following contrast administration (Figure 1). No adjacent cerebral edema was noted. Surgical resection was attempted, but was aborted after a subtotal resection due to hemorrhage. The pathological diagnosis was epithelioid angiosarcoma. Our patient was referred to the University of Alabama at Birmingham for completion of the resection following endovascular embolization of the tumor, which was completed eight months after her initial presentation. Intra-operative findings were significant for a well-capsulized lesion that could be dissected off the dura. No apparent intra-dural extension was noted. The embolization controlled the majority of the vascular supply, resulting in minimal blood loss. At her first post-operative visit, our patient reported improvement in all symptoms, including decreased proptosis, improved vision, and improved left facial numbness. At her latest visit one-and-a-half years after presentation, she was doing well, having completed six weeks of radiation therapy and concurrent bevacizumab. She continued a maintenance phase of bevacizumab for six months after radiation therapy. Her magnetic resonance imaging (MRI) scan remained stable when compared to prior scans.

### Patient 2

**Figure 2:**
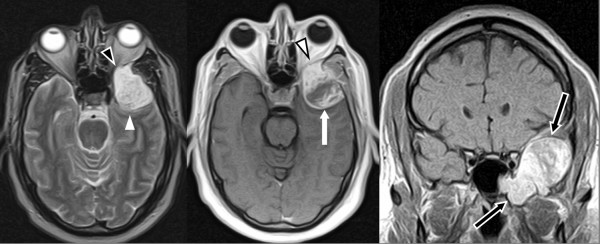
**Axial T2-weighted magnetic resonance image demonstrating a mildly heterogeneously hyperintense mass extending from the left middle cranial fossa (white arrowhead) through the left greater sphenoid wing into the left orbit (outlined black arrowhead).** The axial T1-weighted gadolinium-diethylenetriaminepentaacetic acid (Gd-DTPA)-enhanced image demonstrates identical findings (white arrow and outlined white arrowhead, respectively) and heterogeneous enhancement of the mass. The coronal T1-weighted Gd-DTPA-enhanced image demonstrates the mass extending downward and medially into the pterygoid recess of the left sphenoid sinus (outlined black arrows).

A 47-year-old Caucasian man presented to our facility with acute onset of visual loss in the left eye of 10 days duration. There were also subtle memory problems and word-finding difficulties. Imaging studies demonstrated a large, heterogeneously-enhancing left sphenoid wing mass extending into the sphenoid sinus, thought to be either epidural or dura based and most consistent with meningioma (Figure 2), but with inhomogeneous enhancement and extension into the sphenoid sinus. Intra-operative findings were significant for a highly vascular tumor. Extensive bleeding was encountered until the tumor was removed. The margin of the neoplasm adjacent to the dura could be dissected off the dura without evidence of intra-dural extension. A neuropathological examination demonstrated a high-grade angiosarcoma. Post-operative imaging demonstrated a near-total gross resection, with only a small amount of enhancing tumor remaining in the sphenoid sinus adjacent to the carotid artery. Additional therapy is planned with radiation therapy, temozolomide, and bevacizumab.

**Figure 3:**
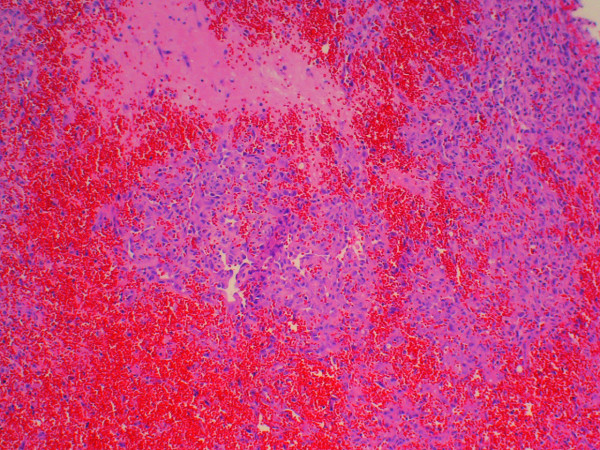
A low-power photomicrograph from the tumor removed from patient 1 revealing a vascular tumor with polygonal cells, spindle cells and abundant hemorrhage (hematoxylin and eosin stain, ×100).

**Figure 4:**
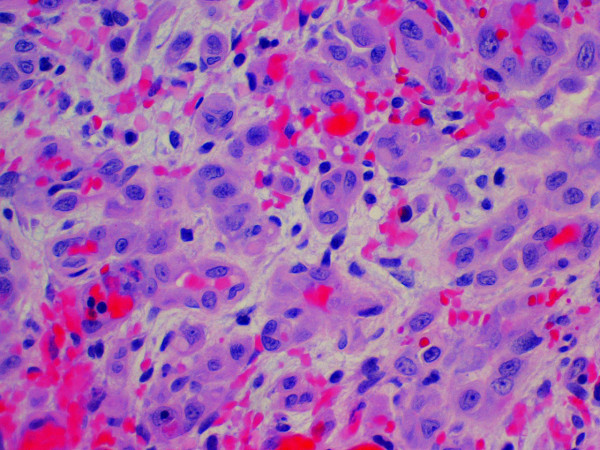
A high-power field of the angiosarcoma from patient 2 showing abortive vascular structures (hematoxylin and eosin stain, ×400).

Microscopic examination of our two cases demonstrate extensive areas of anastomosing vascular channels lined by atypical, plump endothelial cells with enlarged nuclei and prominent nucleoli. These cells are often present in multiple layers or papillary-like tufts projecting into the compressed lumen. Minimal stroma separates the abnormal vessels in these vascular areas (Figures 3 and 4). However, in other areas there are sparse vessels with abundant myxoid stroma within which there are scattered atypical spindle cells arranged singly or in small groups. This latter change was particularly prominent in our second patient’s tumor. Both lesions included areas of hemorrhage and tumor necrosis.

**Figure 5:**
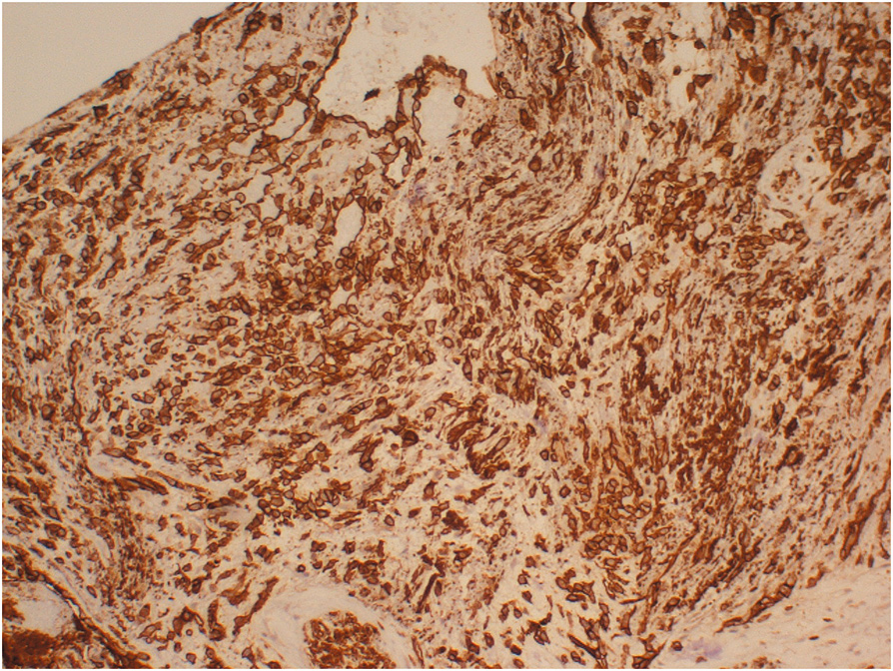
Both tumors were positive for CD31 in the polygonal and spindle cells (CD31 immunoperoxidase stain, ×100).

Immunohistochemical staining for CD31 prominently decorates tumor cells, including the lining cells of the interconnected vascular channels and the spindle cells within the myxoid stroma (Figure 5).

## Discussion

Accurate diagnosis of primary CNS angiosarcoma is greatly enhanced by the recognition of characteristic, though not always specific, features of clinical presentation, imaging characteristics, and histopathology. Usually there is rapid onset of neurological symptoms that relate to the tumor’s location and rapidity of growth [1]. Imaging studies characteristically show a well-demarcated lesion of the cerebral hemisphere with avid enhancement following administration of intravenous contrast material such as gadolinium [5,6]. Although significant vasogenic edema has been reported in prior cases, neither of our patient’s cases were associated with any cerebral edema. Histological examination demonstrates a malignant tumor with a range of differentiation that often includes vascular channels lined by tufted or papillary aggregates of malignant endothelial cells, as well as poorly-differentiated solid areas with malignant spindle cells residing within a collagenous or myxoid stroma [1]. Immunohistochemical staining with CD31, a sensitive and specific endothelial marker, is characteristically positive in both the well-differentiated vasoformative regions and in the less-differentiated solid areas of tumor.

**Figure 6:**
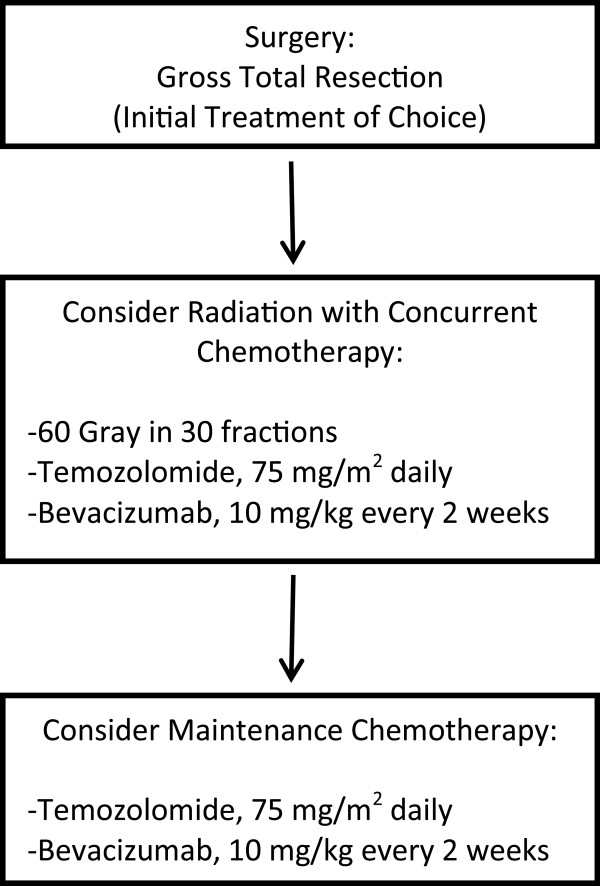
A proposed flow chart for the treatment of intra-cranial angiosarcoma.

In the cases reported over the last 25 years, primary angiosarcoma of the brain is characterized by a high rate of local recurrence and a short median survival time. In the series reported by Mena *et al.*[1], the median survival time in the five patients known to have died was eight months. However, two patients in that study were reported to be alive after follow-up intervals of 39 and 102 months, respectively. The one patient reported by Cookston *et al.*[6] was alive and doing well 42 months following diagnosis, having been treated by gross total resection and adjuvant radiation therapy. While primary therapy is surgical, a few patients, such as this one, have apparently benefited from radiation therapy and/or chemotherapy (Figure 6), either in the adjuvant setting or as a later addition at the time of recurrence.

Chemotherapy for primary CNS angiosarcoma, as for virtually all soft tissue sarcomas, is problematic. Combination chemotherapy has not been shown to be any more effective than single agent therapy in most instances [11]. Temozolomide has shown promising activity against sarcomas in general [11]; it is an option for CNS disease because it crosses the blood brain barrier. For most histological types of sarcoma, single agent therapy with doxorubicin is favored because of its beneficial profile of progression-free survival and relatively tolerable toxicity [10]. Regardless, its use is palliative rather than curative. Paclitaxel has been shown to produce favorable progression-free survival rates at three and six months in both chemonaive and previously-treated patients with the diagnosis of soft tissue angiosarcoma in Phase II clinical trials [12]. Schlemmer *et al.*[13] report a response rate of 62% for paclitaxel in the setting of advanced angiosarcoma of soft tissue. However, the use of paclitaxel or other chemotherapeutic agents has not yet been reported in primary CNS angiosarcoma.

Bevacizumab, a vascular endothelium growth factor (VEGF) inhibitor, has been used with some success in recurrent glioblastoma, a primary CNS tumor that is known to produce VEGF [14]. The use of bevacizumab for primary CNS angiosarcoma has not been reported, although a phase II trial looking at bevacizumab in patients with unresectable soft tissue angiosarcoma is ongoing (NCT00288015). Preliminary results from the first 27 patients in this trial have been briefly reported by Park *et al.*[15]. Median progression-free survival was 12 weeks. These authors also discuss data suggesting that combining temozolomide with bevacizumab may result in a potentiation of the latter’s anti-angiogenic effect.

In addition, two multicenter phase II clinical trials are currently in recruitment to look at paclitaxel and bevacizumab in the treatment of metastatic or unresectable visceral or soft tissue angiosarcoma (NCT01055028 and NCT01303497). These studies do not address primary CNS angiosarcoma; in fact, known CNS disease is an exclusion criterion.

## Conclusions

The rarity of primary angiosarcoma of the brain and the resulting paucity of relevant studies and case reports precludes a definitive judgment concerning optimal therapy. Regardless, suggestions derived from the few published cases, along with the short follow-up from our two more recent cases, may allow the preliminary formulation of a potentially effective treatment plan using modern, multimodality therapy. Surgery with or without tumor embolization, with the goal of gross total resection, remains the standard of care. Post-operative adjuvant radiation therapy may provide a benefit, particularly in cases where gross total resection is not possible. There may be a rationale for using anti-angiogenesis agents such as bevacizumab, perhaps in combination with temozolomide for synergistic anti-angiogenic effect. Finally, treatment results for paclitaxel in soft tissue angiosarcoma suggest that there may also be a role for this chemotherapeutic agent in primary CNS angiosarcoma.

## Consent

Written informed consent was obtained from both patients for publication of this manuscript and any accompanying images. A copy of the written consent is available for review by the Editor-in-Chief of this journal.

## Competing interests

The authors declare that they have no competing interests.

## Authors’ contributions

JRH drafted the manuscript; CAP provided the neuropathology and participated in the final draft; KOR was responsible for the neurosurgical care of both our patients; JKC provided the neuroradiology interpretations for both our patients as well as this section in the manuscript; HMFS was responsible for one of our patient’s chemotherapeutic regimens and helped to draft the manuscript; LBN conceived the study, was responsible for one of our patient’s chemotherapeutic regimens and helped to draft the manuscript. All authors read and approved the final manuscript.
